# Network-based analysis of national strategies for COVID-19 management

**DOI:** 10.1016/j.isci.2025.113907

**Published:** 2025-10-30

**Authors:** Amirreza Salehi, Ardavan Babaei

**Affiliations:** 1 Department of Industrial Engineering, Sharif University of Technology, Tehran, Iran; 2 Faculty of Industrial Engineering, K. N. Toosi University of Technology, Tehran, Iran; 3 Department of Industrial Engineering, Istinye University, Istanbul, Turkey

**Keywords:** Public health, Machine learning

## Abstract

The COVID-19 pandemic highlighted the need for systematic evaluation of national response strategies that account for complex interdependencies across health, economic, and demographic dimensions. This study introduces a network-based framework that integrates machine learning for feature selection, entropy weighting for objective prioritization, and the Analytic Network Process to capture interdependencies among criteria and countries. Analysis of 147 countries shows that testing capacity, demographic structure, and healthcare resilience are decisive in shaping outcomes. High-performing nations, including Finland and Denmark, combined widespread testing with robust infrastructure and stringent public health measures, while resource-constrained countries faced significant challenges. Random Forest importance underscored the role of life expectancy and median age, while entropy weights emphasized testing and mortality-related indicators. Hierarchical clustering revealed regional performance patterns that align with socioeconomic resilience. The findings provide policymakers with a comprehensive tool to design evidence-based interventions, strengthen preparedness, and foster international cooperation in future health crises.

## Highlights

• ML-MCDM framework evaluates COVID-19 responses across 147 countries
• Testing capacity and demographic resilience identified as key performance drivers
• Analytic Network Process captures interdependencies among criteria and countries
• Findings guide policymakers on testing, healthcare, and international cooperation

## Graphical abstract

**Figure:**
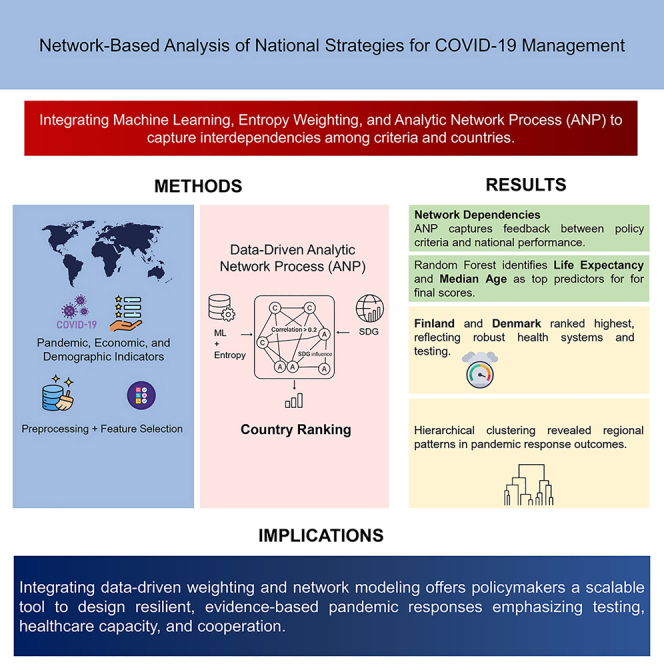

## Introduction

The coronavirus disease 2019 (COVID-19) pandemic, caused by the SARS-CoV-2 virus, emerged in Wuhan, China, in December 2019, swiftly becoming a global health crisis.^1^ As of April 13, 2024, the Worldometer has reported 704,753,890 confirmed cases and 7,010,681 deaths worldwide.^2^ The pandemic has caused major changes in society, affecting different aspects of human life regardless of whether one is infected.^3^^,^^4^ These adjustments include interruptions to usual routines, restrictions on social activities, financial challenges, increased anxiety, and escalated stress levels.^5^ Therefore, governments worldwide have established strict measures to control the spread, such as social distancing, quarantine rules, isolation methods, lockdowns, curfews, and other tactics to reduce transmission from infected to uninfected individuals.^6^^,^^7^ The diverse approaches adopted by various governments in implementing pandemic policies can lead to different outcomes in different countries.

Evaluating the performance of countries’ policies provides valuable insights into identifying the most effective strategies, offering a roadmap for policymakers worldwide in navigating similar pandemic situations. The policymaker’s roadmap can encompass various aspects of countries, including healthcare infrastructure, travel restrictions, economic support, and other pertinent factors.^8^ This study primarily aims to provide policymakers with a comprehensive roadmap for more effectively managing pandemic situations through the performance evaluation of countries and the assessment of their strategies.

Traditionally, performance evaluations have relied on criteria for ranking countries, often overlooking the interconnectedness between these criteria and the countries themselves. However, this paper uniquely considers both the interrelation between criteria and the interconnectedness between countries in its performance evaluation framework. In implementing the performance evaluation, we leverage the integration of machine learning (ML) and multi-criteria decision making (MCDM) techniques.^9^^,^^10^ Based on these motivations, the research addresses the following key questions:

RQ1: In what ways can a network-oriented decision-making framework advance the evaluation of pandemic management by explicitly capturing the complex interdependencies among policy criteria?

RQ2: How can modeling intercountry interdependence reshape our understanding of national performance in a global health crisis and provide more systemic insights compared to traditional isolated evaluations?

RQ3: To what extent can the integration of ML with MCDM enhance methodological rigor by enabling objective feature selection, reducing bias, and strengthening the validity of pandemic response evaluations?

The main contributions of this paper are outlined in the following manner.

1 As of our last knowledge, this study is the first study to consider the network structure in MCDM for COVID-19, utilizing the Analytic Network Process (ANP) method to evaluate performance and reveal the complex connections between important criteria. By utilizing ANP effectively, the framework can successfully determine and assess the connections between different evaluation metrics, leading to a deeper and more thorough grasp of performance dynamics.
2 Going beyond traditional methods, the use of the ANP method now includes examining relationships between countries, providing a comprehensive decision framework that includes the interconnected effects of nations on one another. This comprehensive viewpoint recognizes the complex network of connections that define the worldwide environment, enhancing the assessment process with important insights for making well-informed decisions.
3 Before using ANP, a careful selection of features is made to identify important criteria, guaranteeing the integrity and relevance of the evaluation framework. After conducting the ANP analysis, the framework is enhanced by ML techniques to pinpoint and prioritize the most crucial criteria. This step-by-step method improves the strength of the system and also provides a clear guide for understanding stakeholders, thus helping to make decision-making processes more efficient.

### Literature survey

A rigorous review of the existing literature is essential to position this study within the broader research landscape. This section highlights major prior contributions related to the integration of ML and MCDM in pandemic management and then identifies the remaining gaps that motivate our work.

### Public health evaluation frameworks

A significant stream of research has emerged from established public health frameworks such as the World Health Organization’s Joint External Evaluation (JEE), the COVID-19 Global Evaluation Initiative, the Organisation for Economic Co-operation and Development (OECD) resilience metrics, and the Lancet COVID-19 Commission. These frameworks provide standardized and policy-oriented benchmarks for assessing pandemic preparedness and response. Gupta et al.^11^ proposed a JEE analysis of 55 countries, finding 89.6% of indicators below demonstrated capacity, notably in antimicrobial resistance and biosecurity, with scores correlating to health and socioeconomic factors, though less in the World Health Organization African Region (WHO AFRO). Kayiwa et al.^12^ documented Uganda’s JEE process, highlighting multisectoral participation but also pointing to challenges such as expert dependence, limited government-wide involvement, and difficulties in reducing reliance on external support. While JEE has been widely applied, it relies heavily on self-reporting and static indicators, which restricts its ability to capture dynamic system interactions.

Complementary frameworks have emphasized resilience more explicitly. Zhao et al.^13^ assessed resilience in 60 countries using governance, financing, workforce, and service provision as key dimensions, finding that Switzerland and Japan ranked highest and underscoring governance as a decisive factor for effective response. Similarly, Haldane et al.^14^ examined 28 countries and highlighted governance, financing, workforce capacity, and community engagement as essential pillars of resilience during COVID-19. Arsenault et al.^15^ extended this line of work by studying service disruptions across 10 countries, showing that essential services such as cancer, tuberculosis, HIV, and maternal care were severely impacted and calling for long-term resilience improvements. OECD-focused studies have further demonstrated systemic vulnerabilities. Manavgat and Audibert^16^ evaluated efficiency across 31 OECD countries before and during COVID-19, finding a general decline, with only Estonia and Japan maintaining full efficiency. Together, these works stress resilience, governance, and service delivery as critical, but they largely remain descriptive and do not capture the interdependencies among criteria or mutual influences across countries.

### MCDM approaches in COVID-19 evaluation

Beyond these policy frameworks, numerous studies have used MCDM and ML methods to evaluate pandemic responses. Aydin and Yurdakul^17^ combined data envelopment analysis (DEA) with clustering to classify 142 countries by efficiency, providing useful benchmarking but omitting resilience considerations. Aggarwal et al.^18^ proposed a multi-criterion intelligent decision support system, integrating ML algorithms with susceptible-exposed-infectious-recovered modeling to enhance comparative evaluation of COVID-19 risks. Mete et al.^19^ developed a hybrid MCDM approach that reassessed INFORM risk indicators using the multi-choice best–worst method and Complex Proportional Assessment (COPRAS), yielding scenario-based recommendations for policymakers. Likewise, Xidonas and Steuer^20^ introduced Technique for Order of Preference by Similarity to Ideal Solution (TOPSIS) and Preference Ranking Organization Method for Enrichment Evaluations (PROMETHEE) II for EU COVID-19 evaluation, while Alkan and Kahraman^21^ employed q-rung orthopair fuzzy TOPSIS to prioritize strict quarantine as the most effective response. Magableh and Mistarihi^22^ further demonstrated the adaptability of MCDM by integrating ANP and TOPSIS to assess supply chain resilience, stressing the importance of technological preparedness for future crises.

Additional sectoral studies illustrate the breadth of MCDM applications. Esfahani et al.^23^ applied ANP to rank disruptions in the food supply chain of an Iranian company, identifying labor shortages as the most severe challenge. Oktari et al.^24^ used Strengths, Weaknesses, Opportunities, Threats (SWOT) and ANP to prioritize knowledge management strategies in Indonesia, emphasizing community engagement. Wu et al.^25^ adopted a dynamic network DEA for the airline industry, showing steep efficiency losses during 2019–2020 and partial recovery by 2022. Ozsahin et al.^26^ assessed the pandemic response capacity of 22 countries using MCDM and ranked South Korea highest, emphasizing the robustness of developing countries that adopted evidence-based strategies. While these approaches extend methodological rigor, most still treat criteria and alternatives as independent, thereby overlooking systemic interdependencies that are especially relevant in global crises.

### Data-driven and resilience-oriented models

Recent studies have sought to address these gaps by developing data-driven frameworks that explicitly incorporate resilience and ML. Salehi et al.^27^ introduced the Variant-Informed Decision Support System (VIDSS), which dynamically adapts to COVID-19 variants by combining multi-attribute decision-making with transfer learning. Salehi et al.^28^ proposed a hybrid ML–MCDM framework that explicitly introduced resilience as an evaluation criterion, enabling countries’ ability to recover after peak infection periods to be captured. Another contribution^29^ used AHP and ensemble learning to assess sustainable energy performance, showcasing how subjective judgments can be replaced with data-derived weights for broader applications of MCDM. Meraji et al.^30^ advanced this agenda by evaluating 168 countries with clustering and MCDM methods, introducing “medical waste” as a novel indicator with greater explanatory power than most traditional measures. These emerging frameworks demonstrate the value of combining ML, entropy-based weighting, and advanced MCDM for more objective, scalable, and resilience-oriented evaluations.

### Synthesis and research gap

The reviewed literature highlights several advances but also reveals persistent limitations.

1 First, while established global frameworks such as the WHO’s JEE, OECD resilience assessments, and the Lancet Commission provide standardized benchmarks, they largely rely on static, qualitative, or self-reported indicators. As a result, they fail to capture the complex interconnections between evaluation criteria that shape real-world pandemic dynamics. Ignoring such linkages risks producing misleading rankings and weakening the validity of policy recommendations.
2 Second, most MCDM applications assume independence across alternatives, overlooking the interdependence of countries in the context of a highly contagious global crisis. In practice, the pandemic demonstrated that one nation’s policies—such as border control, vaccination diplomacy, or economic support—can directly affect outcomes in neighboring or partner countries. Failing to model such intercountry influences limits the explanatory power of existing evaluation frameworks.
3 Third, although recent data-driven approaches that integrate ML and MCDM have improved objectivity by reducing reliance on expert judgment, they often focus narrowly on feature selection or ranking exercises without developing comprehensive frameworks that simultaneously address resilience, interdependencies, and methodological robustness. Consequently, there remains a lack of integrated systems that combine effective feature selection, objective weighting methods, and network-based modeling to capture the full complexity of pandemic management.

From a health systems perspective, interdependencies are critical because pandemics operate within complex adaptive systems, where isolated evaluations ignore cascading effects—e.g., a shortfall in health workforce capacity (a WHO building block) not only hampers domestic service delivery but also influences neighboring countries through shared borders or economic ties, amplifying vulnerabilities. This justifies the need for network-based approaches beyond methodological novelty: they align with resilience theory, enabling quantification of how governance capacities like financing and leadership interact to build adaptive responses, as seen in high-performing nations’ ability to leverage international aid. By addressing these gaps, our framework provides a theoretically grounded tool that integrates interdependencies to offer systemic, rather than fragmented, insights into pandemic management.

Addressing these gaps is essential for advancing beyond descriptive or isolated evaluations toward a robust, policy-oriented framework. This study responds to this need by proposing a network-based ML–MCDM model that explicitly incorporates both intercriteria linkages and intercountry influences, thereby providing a more comprehensive and actionable assessment of COVID-19 management performance.

## Results

**Figure 1:**
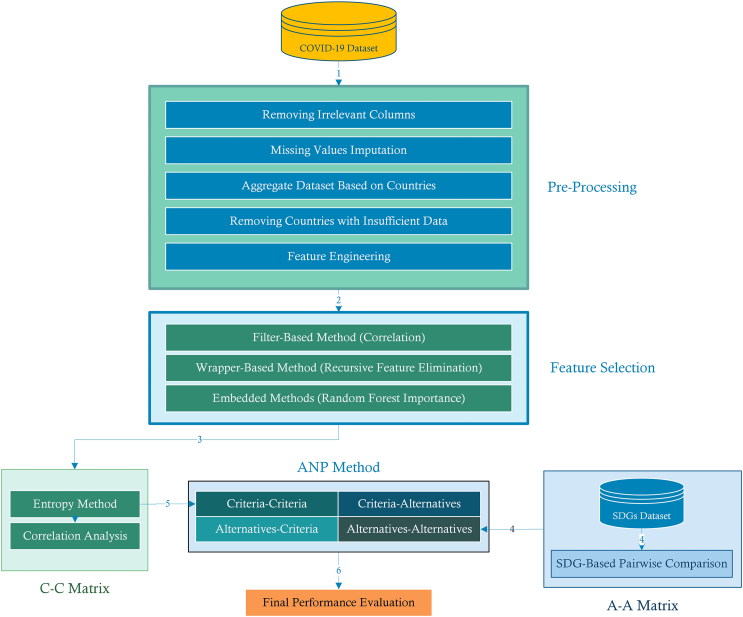
Proposed framework illustration

Using the preprocessed COVID-19 dataset (Table 1) and the selected features identified through various selection methods (Table 2), we applied the proposed analytical framework (Figure 1). The following subsections describe the performance outcomes and comparative results of the developed models.

**Table 1:** Feature descriptions of the preprocessed COVID-19 dataset

Feature	Description
Tests Per Capita	Number of COVID-19 tests conducted per capita
Mortality Rate	Ratio of COVID-19 deaths per capita to COVID-19 cases per capita
Deaths Per Capita	Total number of COVID-19 deaths per capita
Reproduction Rate	Current estimate of the effective reproduction rate (R) of COVID-19
GDP Per Capita	Gross Domestic Product per capita
Life Expectancy	Average life expectancy of the population
Median Age	Median age of the population
Aged 70 Older	Percentage of the population aged 70 and older
Female Smokers	Percentage of female smokers in the population
Population	Total population of the country
Diabetes Prevalence	Prevalence of diabetes in the population
Stringency Index	Index measuring the strictness of government responses to COVID-19
Human Development Index	Composite index measuring average achievement in health, education, and standard of living
Extreme Poverty	Percentage of the population living in extreme poverty
Boosters Per Capita	Number of COVID-19 booster vaccinations administered per capita
Vaccinations Per Capita	Total number of COVID-19 vaccinations administered per capita
Vaccinated Per Capita	Number of individuals who have received at least one dose of a COVID-19 vaccine per capita
Positive Rate	Percentage of COVID-19 tests that are positive
Fully Vaccinated Per Capita	Number of individuals fully vaccinated against COVID-19 per capita
Cardiovascular Death Rate	Death rate due to cardiovascular diseases
Tests Per Case	Number of COVID-19 tests conducted per confirmed case
Aged 65 Older	Percentage of the population aged 65 and older

**Table 2:** Selected features under different feature selection methods and majority vote

Features	Correlation	RFE	Random Forest	Selected
Tests Per Capita	✔	✔	✔	✔
Mortality Rate	✔	✔	✔	✔
Deaths Per Capita	✔	✔	✔	✔
Reproduction Rate	✔	✔	✖	✔
GDP Per Capita	✔	✖	✔	✔
Life Expectancy	✔	✖	✔	✔
Median Age	✔	✖	✔	✔
Aged 70 Older	✔	✔	✔	✔
Female Smokers	✔	✖	✖	✖
Population	✖	✖	✔	✖
Diabetes Prevalence	✖	✔	✖	✖
Stringency Index	✖	✖	✔	✖
Human Development Index	✔	✔	✔	✔
Extreme Poverty	✔	✖	✖	✖
Boosters Per Capita	✔	✔	✖	✔
Vaccinations Per Capita	✔	✔	✖	✔
Vaccinated Per Capita	✔	✔	✖	✔
Positive Rate	✖	✔	✔	✔
Fully Vaccinated Per Capita	✔	✔	✖	✔
Cardiovascular Death Rate	✔	✖	✖	✖
Tests Per Case	✖	✔	✔	✔
Aged 65 Older	✔	✖	✔	✔

### Feature weighting using the entropy method

The analysis of COVID-19 response effectiveness across various countries was conducted using multiple methods, including the entropy method for feature weighting, ANP for deriving country scores, and hierarchical clustering for pattern identification. The comprehensive evaluation aimed to uncover the key determinants of effective pandemic management and provide insights into country-specific performance. By integrating diverse factors such as healthcare infrastructure, economic status, demographic characteristics, and public health measures, this study offers a multi-faceted perspective on global responses to COVID-19. The following sections present detailed findings, including the significance of different features, the convergence process of the supermatrix, comparative performance scores of countries, and the clustering of countries based on their response effectiveness.

The entropy method utilized in this analysis provided a nuanced understanding of the factors that contribute to the effectiveness of COVID-19 responses. Among the evaluated features provided in the Table 3, “Tests Per Capita” emerged as the most crucial, emphasizing the importance of widespread testing in identifying and tracking infections. Additionally, indicators such as “Deaths Per Capita” and “Mortality Rate” received significant weights, underlining the critical need to minimize fatalities. Economic factors, including “GDP Per Capita,” also played a substantial role in assessing response effectiveness. Interestingly, vaccination metrics were found to have moderate weights in the analysis. “Fully Vaccinated Per Capita” and “Vaccinations Per Capita” were less influential compared to testing and mortality indicators, indicating that while vaccination is essential, a holistic approach that includes testing and case management is crucial in pandemic response efforts. Demographic factors like “Aged 70 Older” and “Median Age” held moderate weight in the analysis, highlighting their influence on vulnerability to severe illness. Metrics such as “Reproduction Rate” and “Positive Rate” received relatively low weights, which could be attributed to the specific dataset used or the time frame of the analysis. The entropy-derived weights offer valuable insights for policymakers in prioritizing effective strategies for addressing COVID-19. By focusing on testing, mortality reduction, economic considerations, and population vulnerability, policymakers can tailor response efforts to better combat the pandemic and protect public health.

**Table 3:** Weights assigned to features using the entropy method

Feature	Weight
Tests Per Capita	0.178980
Deaths Per Capita	0.089226
GDP Per Capita	0.080313
Mortality Rate	0.066016
Boosters Per Capita	0.068741
Cases Per Capita	0.105245
Vaccinations Per Capita	0.032250
Life Expectancy	0.016691
Fully Vaccinated Per Capita	0.020015
Human Development Index	0.022314
Aged 70 Older	0.058901
Median Age	0.034706
Tests Per Case	0.081987
Vaccinated Per Capita	0.017125
Reproduction Rate	0.015190
Positive Rate	0.056499
Aged 65 Older	0.055802

### Convergence of the supermatrix in ANP analysis

**Figure 2:**
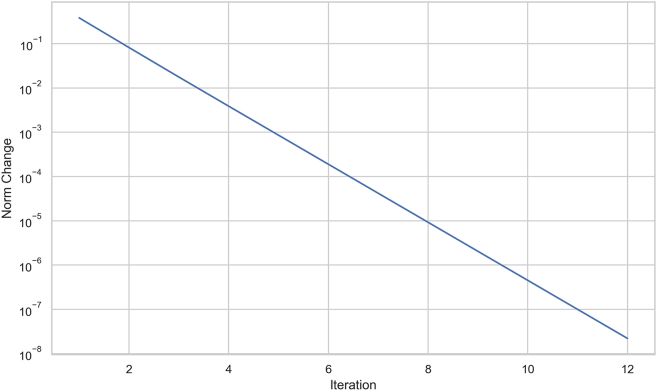
Convergence of the supermatrix

In solving the supermatrix, we set the tolerance to 1e-9 and maximum iterations to 10,000. Convergence was achieved after 12 iterations. Figure 2 provides an illustration of the norm changes over the iterations. Norm changes refer to the differences between successive iterations of the matrix multiplication process, measured using the Euclidean norm. The plot is essential as it visually demonstrates the stabilization of matrix values, with significant changes initially that diminish over time, approaching zero. This rapid decline confirms the effective convergence of the supermatrix, ensuring that the ANP analysis results are stable and reliable. This visualization helps assess the efficiency of the convergence process, providing confidence in the robustness of the final results.

### Comparative country performance based on ANP scores

**Figure 3:**
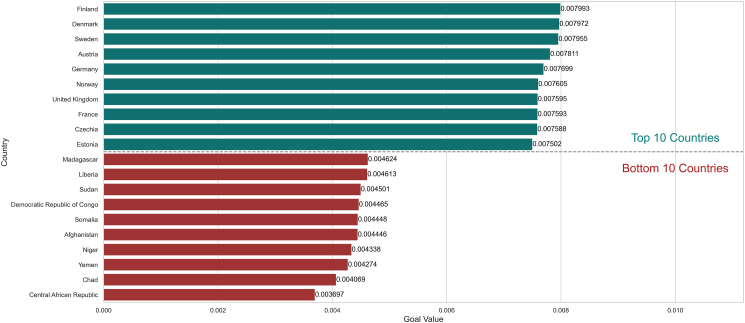
Comparative performance scores of the top 10 and bottom 10 countries using the ANP framework

Figure 3 illustrates the comparative performance scores of the top 10 and bottom 10 countries in managing the COVID-19 pandemic using the ANP framework. Finland leads with the highest score of 0.007993, demonstrating exceptional effectiveness in pandemic response measures. Denmark and Sweden follow with scores of 0.007972 and 0.007955, respectively, highlighting their robust health infrastructure and proactive policies. Austria and Germany also feature in the top 10, underscoring their advanced healthcare systems and efficient resource allocation. In contrast, the bottom 10 countries, including the Madagascar (0.004624) and Liberia (0.004613), exhibit significantly lower scores, reflecting challenges in healthcare infrastructure, economic constraints, and limited access to essential resources. The stark contrast between the top and bottom performers emphasizes the crucial role of comprehensive policy implementation, international cooperation, and sustainable development in pandemic management. This analysis provides valuable insights for policymakers to identify effective strategies and areas needing improvement, ensuring better preparedness for future health crises. The more detailed rank of countries is provided in Table S1.

### Hierarchical clustering of countries based on response effectiveness

**Figure 4:**
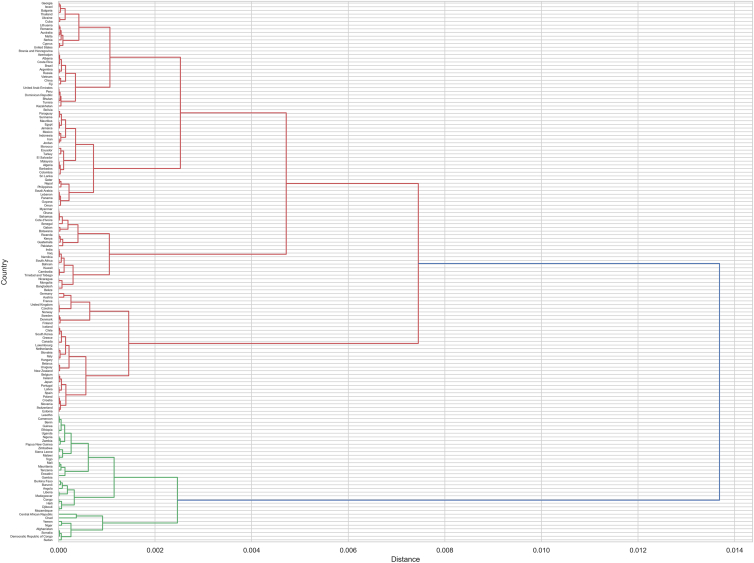
Hierarchical clustering dendrogram

Figure 4 provides a hierarchical clustering dendrogram to illustrate the grouping of countries based on their scores. This chart visually represents the similarity between countries, allowing us to see which countries are more similar and form clusters. The hierarchical clustering method used is Ward’s method, which minimizes variance within each cluster for well-separated groups. The Euclidean distance metric measures the dissimilarity between countries. The dendrogram identifies distinct clusters of countries with similar characteristics, offering insights into patterns and relationships. Hierarchical clustering with Ward’s method and Euclidean distance is chosen for effective grouping and clear separation. The dendrogram is truncated to display the top 30 clusters for readability. The dendrogram reveals regional and socioeconomic similarities in COVID-19 management performance among countries, with clusters indicating shared policy effectiveness. This suggests that policymakers should encourage benchmarking and collaboration within clusters to adopt successful strategies and share knowledge. Additionally, resource allocation and interventions can be tailored to the specific needs of each cluster, improving overall pandemic response efficiency and effectiveness. For decision-makers, this means focusing support and resources on clusters with similar challenges to ensure efficient and impactful global aid distribution.

Feature importance analysis is pivotal in understanding the relative contribution of each factor in predicting the ANP-derived scores for the performance evaluation of countries in managing the COVID-19 pandemic. By identifying which features have the most significant impact on the model’s predictions, policymakers can better prioritize resources and strategies to enhance pandemic response effectiveness. In this study, the Random Forest algorithm was chosen for its robustness and ability to handle complex interactions between features. Random Forest is an ensemble learning method that combines multiple decision trees to improve predictive accuracy and control over-fitting. Its inherent feature importance metric provides an intuitive understanding of each variable’s influence on the target variable. The data preprocessing involved splitting the dataset into features and target variables, followed by normalizing the features using StandardScaler. This standardization ensures that all features contribute equally to the model training process. The dataset was then divided into training and testing sets with an 80-20 split, ensuring that the model could be evaluated on unseen data. The Random Forest model was trained using the training set and evaluated using the test set. The R-squared value of 87% indicates that the model explains a substantial proportion of the variance in the ANP-derived scores, demonstrating its predictive power. Although the ANP-derived scores are constructed from the same set of features, applying Random Forest provides complementary insights by quantifying the marginal contribution and nonlinear interactions of individual predictors in shaping the composite outcomes. In this way, Random Forest does not simply replicate the ANP process but helps disentangle which features drive score variability most strongly, offering an additional layer of interpretability.

### Feature importance analysis

**Figure 5:**
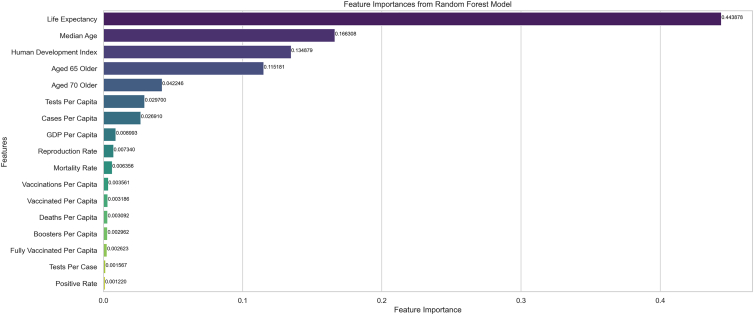
Importances of features in predicting ANP-derived scores

The feature importance analysis revealed several key insights, as presented in Figure 5. The most crucial determinant was Life Expectancy (0.443878), indicating that countries with higher life expectancy are better equipped to manage the pandemic. This could be attributed to better overall health infrastructure and a healthier population, essential for effective pandemic response. Median Age (0.166308) also underscored the impact of demographic factors on pandemic management, as countries with a younger median age may have a lower burden of severe cases and mortality, facilitating better overall performance. The high importance score of the Human Development Index (HDI, 0.134879) highlighted the role of socioeconomic development in managing health crises. Nations with higher HDI likely have better healthcare systems, education, and economic resources, all contributing to more effective pandemic responses. The proportion of older adults in a population was another critical factor, as evidenced by the importance of Aged 65 Older (0.115181) and Aged 70 Older (0.042246). Older populations are more vulnerable to severe outcomes, necessitating targeted healthcare strategies. The moderate importance of Tests Per Capita (0.029700) emphasized the need for widespread testing to control and monitor the virus’s spread effectively. Additionally, the number of Cases Per Capita (0.026910) reflected the infection rate within a country, influencing its overall response strategy and effectiveness. While GDP Per Capita (0.008993) was less significant than other features, it still played a role, indicating that economic resources contribute to a country’s ability to respond to the pandemic. Features such as Reproduction Rate (0.007340) and Mortality Rate (0.006356) provided insights into the dynamics of virus spread and fatality rate, both crucial for shaping response measures. Interestingly, vaccination-related features such as Vaccinations Per Capita (0.003561), Fully Vaccinated Per Capita (0.002623), and Boosters Per Capita (0.002962) had lower importance scores, suggesting that while vaccination is essential, its impact relative to other features in this model was less pronounced. Similarly, Tests Per Case (0.001567) and Positive Rate (0.001220) had minimal influence, possibly due to the comprehensive nature of other more significant factors.

## Discussion

This study presents a framework integrating ML and MCDM to evaluate national COVID-19 management performance across 147 countries, employing feature selection, entropy-based weighting, the ANP, and hierarchical clustering. The findings offer a systemic assessment of pandemic responses, capturing interdependencies among criteria and countries, and provide actionable insights for policymakers to strengthen resilience and effectiveness in global health crises. This section interprets the results with precision, acknowledges the model’s static nature while addressing temporal dynamics, and delineates evidence-based managerial implications, ensuring alignment between model outputs and policy recommendations.

### Interpretation of findings

The framework analyzes a preprocessed dataset encompassing 147 countries and 22 features, including health metrics, economic indicators, and vaccination statistics. Entropy-based weighting prioritizes criteria with substantial variability, notably Tests Per Capita and Reproduction Rate, reflecting diverse national testing strategies and transmission control efforts. Vaccination-related indicators, such as Vaccinations Per Capita and Fully Vaccinated Per Capita, received lower weights due to widespread global vaccination coverage by 2023–2024 and reduced efficacy against infection from variants like Omicron, diminishing their discriminative power. Random Forest feature importance identifies Life Expectancy and Median Age as primary predictors, underscoring the pivotal role of demographic and health resilience in shaping pandemic outcomes.

ANP rankings highlight top performers which excel in testing capacity, robust public health measures, and advanced healthcare infrastructure. Conversely, lower-ranked countries face challenges in these areas, compounded by limited socioeconomic resilience, as indicated by Sustainable Development Goals (SDG) scores. Hierarchical clustering reveals distinct patterns, with high-performing clusters, predominantly in Europe, characterized by strong healthcare systems and stringent policies, while lower-performing clusters, often in Sub-Saharan Africa, reflect resource and governance constraints.

### Managerial implications

The findings translate into actionable policy recommendations, each anchored to model outputs—entropy weights, Random Forest importance scores, ANP rankings, and clustering patterns—to ensure coherence and deliver practical guidance beyond descriptive statistics. These recommendations address critical dimensions of pandemic management, informed by the interdependencies modeled in the ANP framework.

#### Strengthen testing infrastructure

The high entropy weight for Tests Per Capita highlights its role in distinguishing performance. Top-ranked countries, such as Finland and Denmark, implemented extensive testing early, enabling rapid case detection and containment. Policymakers should prioritize scalable, accessible testing infrastructure, particularly in resource-constrained settings like Chad, to enhance early response capabilities.

#### Target mortality reduction strategies

The prominence of mortality rate in entropy weights and ANP interdependencies emphasizes protecting vulnerable populations. High-performing countries reduced mortality through targeted interventions, such as prioritizing elderly care, supported by the significant Random Forest importance of Median Age. Tailored measures, including early treatment protocols, are essential to minimize fatalities.

#### Enhance economic resilience

Despite moderate Random Forest importance for GDP Per Capita and HDI, their influence in ANP rankings underscores economic strength in top performers like Sweden and Germany. Economic support mechanisms, such as subsidies or income relief, mitigate disruptions, enabling sustained public health compliance, particularly in lower-ranked countries like Yemen.

#### Tailor interventions to demographic profiles

The high Random Forest importance of Life Expectancy and Median Age indicates demographic influences on outcomes. Countries with older populations, such as Japan, implemented protective measures for the elderly, reducing mortality. Interventions should align with demographic characteristics to ensure robust support for vulnerable groups.

#### Invest in healthcare capacity

The dominant Random Forest importance of life expectancy reflects healthcare infrastructure’s critical role. High-ranking countries like Norway and Austria invested in hospital beds and medical personnel, enhancing response capabilities. Long-term healthcare investments are vital, especially for low-ranked countries like the Central African Republic.

#### Sustain vaccination as a core strategy

Although vaccination indicators received lower entropy weights and Random Forest scores due to high global coverage and variant-driven efficacy declines, their role in reducing severe outcomes remains critical. Top performers integrated vaccination with testing and public health measures. Policymakers should address vaccine hesitancy, ensure booster accessibility, and adapt to variant-driven challenges.

#### Implement robust public health measures

The high entropy weight for reproduction rate underscores transmission control through measures like mask mandates and social distancing. Clustering patterns indicate high-performing countries enforced such policies effectively. Flexible, evidence-based measures are crucial, particularly in densely populated or resource-limited settings.

#### Foster international cooperation

The ANP’s alternatives-to-alternatives (A-A) matrix, using SDG scores, highlights intercountry influences, such as aid from high-SDG nations like Germany to lower-SDG ones like Ethiopia. Clustering patterns suggest regional cooperation enhances outcomes. Policymakers should strengthen global partnerships and update preparedness plans for future pandemics, incorporating lessons from variant-driven shifts.

### Future directions

The model’s static aggregation up to April 2024 limits its ability to capture temporal dynamics, such as policy adjustments or declining vaccine efficacy against infection with variants like Omicron, though protection against hospitalization persisted. Future research could incorporate dynamic models, such as generalized additive models or time-series network analysis, to reflect evolving policy and epidemiological trends. Integrating real-time data or region-specific variant profiles could further refine insights. Coherence between model outputs and recommendations is ensured by anchoring each implication to specific findings: entropy weights prioritize testing and transmission control, Random Forest scores emphasize demographic and healthcare factors, ANP rankings reflect holistic performance, and clustering identifies regional patterns. These insights extend beyond descriptive statistics, offering policymakers a robust framework to optimize responses to current and future pandemics.

In summary, the COVID-19 pandemic posed unprecedented challenges to global public health systems, necessitating robust frameworks to evaluate national management strategies and identify effective policies. Traditional evaluation methods often overlook the complex interdependencies among criteria and countries, limiting their ability to capture the interconnected nature of global health crises. This study addresses this problem by developing an integrated ML and MCDM framework to assess the performance of 147 countries, utilizing feature selection, entropy-based weighting, the ANP, and hierarchical clustering.

The framework reveals that high-performing countries, such as Finland and Denmark, excel due to extensive testing, robust healthcare infrastructure, and stringent public health measures, as evidenced by high entropy weights for Tests Per Capita and Reproduction Rate. In contrast, lower-ranked countries, such as the Central African Republic and Chad, face challenges stemming from resource constraints, reflected in their SDG scores. Random Forest importance scores underscore the pivotal role of Life Expectancy and Median Age, highlighting demographic and health resilience as key determinants of outcomes. Hierarchical clustering identifies regional performance patterns, guiding targeted interventions.

Central to this study’s contribution is the ANP’s network-based approach, which uniquely models criteria-to-criteria (C-C) interdependencies, such as testing influencing mortality rates, and A-A interdependencies, capturing intercountry influences like economic aid or policy diffusion. By integrating data-driven entropy weights and Random Forest feature selection, the framework replaces subjective judgments with empirical rigor, offering a scalable tool for evaluating complex systems. These findings advocate for balanced strategies combining testing, economic support, and public health measures to enhance resilience, providing policymakers with actionable insights for current and future pandemics. This approach sets a new benchmark for performance evaluation, advancing the field through its comprehensive modeling of interconnected global dynamics.

Beyond the measurable health and economic indicators, pandemic performance is also shaped by broader systemic dynamics such as financing flows, governance quality, social trust, equity in resource distribution, and the political context of decision-making. Though excluded from our current model, these factors critically influence the effectiveness of technical measures. Future extensions could integrate such qualitative and institutional dimensions to provide a more comprehensive understanding of resilience in global health crises.

### Limitations of the study

This study evaluates COVID-19 management strategies using a network-based ML–MCDM framework that integrates interdependencies across criteria and countries. While the approach enhances systemic insights, several limitations remain. First, the analysis is based on aggregated, static data up to April 2024, which may not fully capture evolving dynamics such as variant emergence, policy shifts, or temporal changes in vaccination efficacy. Second, proxy indicators such as SDG scores were used to model intercountry interdependencies, which may not reflect real-time cross-border influences or informal cooperation mechanisms. Third, the framework emphasizes quantitative indicators and does not incorporate qualitative dimensions of pandemic response, including governance quality, social trust, and political context, which are critical to resilience. Finally, while the findings offer policy guidance, their applicability may vary across regions due to differences in data reliability, reporting practices, and contextual constraints.

## Resource availability

### Lead contact

Requests for further information and resources should be directed to and will be fulfilled by the lead contact, Amirreza Salehi (sar.salehiamiri@ie.sharif.edu).

### Materials availability

This study did not generate new biological, chemical, or physical materials. All analyses were computational and conducted on publicly available secondary datasets.

### Data and code availability

• Primary raw data used in this study are publicly available from Our World in Data (COVID-19 datasets) and the SDG indices (2020–2023).
• Processed data tables, analysis scripts, and code used to generate the results and figures in the manuscript are available from the lead contact upon reasonable request.

## STAR★Methods

### Key resources table

**Table:** 

REAGENT or RESOURCE	SOURCE	IDENTIFIER
**Deposited data**		

COVID-19 global cases, testing, mortality, vaccination, and demographic indicators (country-level, 2020–2024)	Our World in Data	https://ourworldindata.org/coronavirus
Sustainable Development Goals (SDG) country indices, 2020–2023	United Nations Sustainable Development Report	https://dashboards.sdgindex.org/

**Software and algorithms**		

Python (Version 3.9+)	Python Software Foundation	https://www.python.org/
Jupyter Notebook	Project Jupyter	https://jupyter.org/

### Experimental model and study participant details

This study did not involve human or animal subjects. The experimental unit was at the country level, with 147 countries as “participants.” Each country was represented by demographic, economic, and health-related features derived from secondary public datasets.

### Method details

This section describes the methodological framework for evaluating national COVID-19 management performance, addressing the limitations of traditional evaluation methods that assume independence of criteria and alternatives, such as TOPSIS or the Analytic Hierarchy Process. These methods fail to capture the interdependencies among policy criteria and countries prevalent in a global health crisis. The proposed framework integrates ML for feature selection, the Entropy method for criteria weighting, and the Analytic Network Process for network-based decision-making to provide a data-driven evaluation of pandemic response effectiveness. The approach models complex interactions, such as the influence of testing rates on mortality or cross-border policy effects, to inform strategic decision-making.

The following subsections present the foundational details of the Entropy method and the Analytic Network Process, ensuring clarity in the integrated framework. The methodology transforms preprocessed data into country performance rankings through a systematic process, depicted in Figure 1, which clarifies the data-driven derivation of entropy weights from the normalized decision matrix.

#### Entropy method

The Entropy Method is a quantitative technique used to measure the amount of disorder or uncertainty within a system. It is widely applied in various fields, including information theory, decision-making, and statistics, to assess the distribution and significance of data. In the context of decision-making, the Entropy Method helps in determining the weight of each criterion by evaluating the variability of the data. The Entropy for a given criterion j is calculated as follows:

1 Normalize the values of each criterion to obtain a proportion $p_{ij}$:

(Equation 1) $$p_{ij}=\frac{x_{ij}}{\sum_{i=1}^{n}x_{ij}}$$

where $x_{ij}$ represents the value of criterion j for observation i, and n is the total number of observations.

2 Calculate the entropy value $E_{j}$ for each criterion j:

(Equation 2) $$E_{j}=-k\sum_{i=1}^{n}p_{ij}\;\ln\left(p_{ij}\right)$$

where k is a constant equal to *n*$\frac{1}{\ln\left(n\right)}$.

3 Determine the degree of diversification $d_{j}$ for each criterion j:

(Equation 3) $$d_{j}=1-E_{j}$$

4 Compute the weight $w_{j}$ for each criterion j:

(Equation 4) $$w_{j=}\;\frac{{\mathrm{d}}_{j}}{\sum_{j=1}^{m}{\mathrm{d}}_{j}}$$

where m is the total number of criteria.

Entropy method assigns higher weights to criteria with greater variability, indicating their higher importance in the decision-making process.

#### ANP method

ANP is a decision-making technique tailored for intricate decision problems and networks. It serves as a robust tool for constructing decision support systems.^31^ ANP extends the capabilities of AHP by addressing the challenges posed by interdependencies among variables within a system. This is achieved by segmenting variables into distinct decision criteria and nested sub-criteria.^32^ The ANP employs a feedback-driven approach for problem modeling, rather than relying solely on a hierarchical structure for problem-solving.^33^ The ANP approach is particularly useful in complex decision-making scenarios where interdependencies among criteria and alternatives exist. It captures both the direct and indirect influences among components, leading to more robust and realistic decision outcomes. The general steps in ANP are as follows.

1 First, problem definition and network construction involves clearly defining the problem and identifying the goal, criteria, and alternatives. This step includes constructing a network model where clusters (criteria and alternatives) are represented as nodes, and the dependencies among them are represented as edges.
2 Next, pairwise comparisons and priority vectors require conducting pairwise comparisons for elements within the same cluster and between different clusters. These comparisons use a scale of relative importance to generate a set of priority vectors for each comparison matrix.
3 Following this, supermatrix formation arranges the priority vectors into a supermatrix, which represents the relationships and influences among the elements. The supermatrix is composed of submatrices that show the influence of each element on every other element.
4 In the weighted supermatrix step, the supermatrix is converted into a weighted supermatrix by multiplying each column by the corresponding cluster weight, ensuring that the matrix is column-stochastic (each column sums to one).
5 The limit supermatrix is then obtained by raising the weighted supermatrix to powers until it converges to a steady state, representing the long-term stable influences among the elements.
6 Finally, in the synthesis of results, the priority weights of the alternatives are extracted from the limit supermatrix. These weights indicate the overall ranking or performance of the alternatives concerning the goal.

For more detailed information on the ANP methodology and its application, readers can refer to.^34^

#### Preprocessing

The dataset used as the foundation for this paper is sourced from Our World in Data. To ensure the quality and reliability of our dataset, we conducted rigorous preprocessing steps. Initially, we identified and removed irrelevant columns that did not contribute substantively to our analysis. Next, we addressed missing data issues, particularly focusing on temporal features. For these variables, we employed a method involving monthly averages computed via a 30-day rolling mean, complemented by linear interpolation for residual gaps in the data. Static features like GDP Per Capita were imputed using country-specific means where data was missing, with global mean values applied where necessary. Subsequently, we aggregated the dataset by location, utilizing mean aggregation for most features, while adopting sum aggregation for critical metrics including new cases, death cases, test cases, and various vaccination-related indicators. This aggregation approach culminated in a consolidated summary DataFrame, facilitating comprehensive analysis across geographical regions.

Countries with incomplete data in essential metrics such as Total cases, Total deaths, and Total tests were systematically excluded to maintain data integrity. Moreover, nations lacking information on Vaccination-related features were also omitted from subsequent analyses. For countries with missing values in People Fully Vaccinated and Total Boosters, we developed estimation procedures based on the ratio of these metrics to Total vaccination, ensuring robustness in our dataset’s completeness and accuracy. Equations 1, 2, 3, 4, 5, and 6 outline the procedure for estimating the missing values in the People Fully Vaccinated and Total Boosters data.

(Equation 5) $$P_{fv/tv}=\frac{\text{People}\;\text{Fully}\;\text{Vaccinated}}{\text{Total}\;\text{Vaccination}}$$

(Equation 6) $${\bar{P}}_{fv/tv}=\frac{1}{n}\sum_{i=1}^{n}P_{fv/tv}\left(i\right)$$

(Equation 7) $$\text{People}\;\text{Fully}\;\text{Vaccinate}d_{\text{imputed}}={\text{Total}\;\text{Vaccination}\times \bar{P}}_{fv/tv}$$

(Equation 8) $$P_{tb/tv}=\frac{\text{Total}\;\text{Boosters}}{\text{Total}\;\text{Vaccination}}$$

(Equation 9) $${\bar{P}}_{tb/tv}=\frac{1}{n}\sum_{i=1}^{n}P_{tb/tv}\left(i\right)$$

(Equation 10) $${\text{Total}\;\text{Boosters}}_{\text{imputed}}={\text{Total}\;\text{Vaccination}\times \bar{P}}_{tb/tv}$$

In refining our dataset for further analysis, we transformed population-related features by recalculating them on a per capita basis. This adjustment provides a normalized perspective, essential for comparative analyses across countries of varying population sizes. Additionally, we computed the mortality rate as an auxiliary feature, augmenting our dataset with an important health-related metric for deeper insights into the pandemic’s impact on global populations. The calculation of Mortality Rate can be determined in the following manner.

(Equation 11) $$\text{Mortality}\;\text{Rate}=\frac{\text{Death}\;\text{Per}\;\text{Capita}}{\;\text{Cases}\;\text{Per}\;\text{Capita}}$$

These preprocessing and feature engineering steps are foundational to our subsequent analyses, ensuring that our dataset is both comprehensive and robust for investigating the dynamics of COVID-19 and governmental responses across diverse national contexts.

#### Dataset description

This section provides an overview of the dataset after preprocessing. The dataset consists of comprehensive information on various features from multiple countries.^35^^,^^36^^,^^37^ After rigorous data cleaning and feature engineering, the final dataset includes 22 features across 147 countries. These features encompass a range of indicators such as health metrics, economic indicators, and vaccination statistics. Detailed descriptions of each feature can be found in Table 1.

#### Feature selection

Feature selection plays a crucial role in enhancing the interpretability, efficiency, and generalizability of predictive models by identifying the most relevant predictors while reducing noise and overfitting.^38^ In our study, we employed a comprehensive approach encompassing three main categories of feature selection methods: filter-based, wrapper-based, and embedded methods.

1 Correlation, as a Filter-Based method, evaluates the statistical connection between each feature and the target variable. In this paper, features with correlation coefficients above 20% are considered to be more predictive. We opted for this approach because of its simplicity and speed in pinpointing potentially important predictors in extensive datasets.
2 Recursive Feature Elimination (RFE), a Wrapper-Based technique, eliminates features one by one and evaluates their effect on model performance in order to find the best subset.^39^ In this approach, the optimal number of features is determined by selecting the number that minimizes the Mean Squared Error (MSE). Based on the results, we have chosen to use 14 features as parameters for the RFE method. This approach was chosen for its ability to assess subsets of features using predictive accuracy, potentially capturing nonlinear relationships and interactions between variables.
3 Random Forest Importance as Embedded Methods Utilizing the Random Forest algorithm, this method evaluates feature importance based on how much each feature contributes to decreasing impurity within decision trees.^40^ A threshold of 0.01 for random forest importance is chosen to select features that make a substantial contribution to reducing impurity, thereby enhancing model efficiency and interpretability. Random Forest was chosen for its ability to handle complex interactions and nonlinearities in the data, making it suitable for capturing nuanced relationships among predictors.

We opted for these specific methods to ensure robust feature selection tailored to our dataset’s characteristics. Correlation provides a quick initial assessment of feature relevance, RFE refines the feature subset by considering their collective impact on model performance, and Random Forest captures both individual and collective feature importance within a predictive model framework. To finalize the feature selection process, we employed a majority vote strategy. This approach aggregates the selections made by each individual method (Correlation, RFE, and Random Forest) to identify features consistently deemed important across different methodologies. This ensures a balanced consideration of both statistical relationships and predictive power in our feature subset. Table 2 provides a summary of selected features identified by each method and their total votes under the majority vote approach.

This structured approach to feature selection enhances the robustness and interpretability of our predictive models, laying a solid foundation for subsequent analyses of COVID-19 dynamics and policy responses across diverse geographical contexts.

#### Integrated decision-making framework

The proposed framework combines ML-based feature selection, Entropy-based criteria weighting, and the Analytic Network Process to transform preprocessed data into country performance rankings. This integration captures interdependencies among criteria and alternatives, enabling a comprehensive evaluation of COVID-19 management strategies. The Analytic Network Process models these relationships as a network with feedback loops, distinguishing it from methods like TOPSIS or the Analytic Hierarchy Process, which assume independence and overlook dynamics such as cross-border transmission or policy diffusion. To ensure objectivity, the framework replaces subjective pairwise comparisons with Entropy weights for criteria interdependencies and Sustainable Development Goals scores for country interdependencies. This approach quantifies influences based on empirical data variability and socioeconomic resilience, providing robust insights for pandemic response.

##### Methodological components

The framework integrates three components to overcome limitations in traditional evaluations. ML selects relevant criteria through correlation analysis, Recursive Feature Elimination, and Random Forest importance, ensuring unbiased identification of predictors. The Eentropy method assigns weights based on data variability, prioritizing features with higher dispersion as more informative for decision-making. The Analytic Network Process structures criteria and countries as interconnected clusters, capturing feedback such as the influence of testing capacity on mortality rates or one country’s resilience on another’s through aid or trade.

In the context of COVID-19 response activities, each method contributes distinct insights into effectiveness. ML feature selection identifies predictors like Tests Per Capita, which enable early detection and reduce transmission, as seen in high-performing countries with robust testing regimes. Entropy weighting quantifies criteria importance by variability, prioritizing Reproduction Rate to assess containment strategies’ success in lowering infection spread. The Analytic Network ProcessANP then models these as a network, evaluating how interdependencies—such as vaccination impacting reproduction rates or economic stability influencing healthcare access—drive overall response effectiveness, providing a holistic view of policy outcomes.

The Analytic Network Process is selected for its capacity to model interdependencies essential in pandemics, unlike hierarchical methods that ignore mutual influences. Entropy weights measure the relative importance and influence of criteria, replacing subjective judgments with objective quantification derived from the decision matrix. Sustainable Development Goals scores proxy country interdependencies, reflecting factors like healthcare and economic stability that shape mutual pandemic impacts. This substitution enhances reproducibility and aligns the framework with standardized data.

The Sustainable Development Goals (SDG) index, aggregating scores from 2020 to 2023,^41^ serves as a proxy for country interdependencies by capturing socioeconomic resilience factors, such as health system capacity and economic stability, which shape mutual influences during a pandemic. High-SDG countries, with robust healthcare or economic resources, often lead regional coordination, share aid, or influence policy diffusion, impacting neighbors’ COVID-19 responses. While static, the SDG index reflects structural capabilities that mediate contagion dynamics indirectly, making it a suitable, data-driven input for the Alternatives-to-Alternatives matrix, replacing subjective judgments with standardized metrics.

##### Data transformation and matrix construction

The framework transforms data into rankings through a five-step process, which illustrates the flow from preprocessing to ranking and confirms entropy’s derivation from the normalized decision matrix.

1 Decision Matrix Normalization: The preprocessed dataset forms a decision matrix $=\left[x_{ij}\right]$, with countries as rows i=1,…,m and criteria as columns j=1,…,n normalized to a [0,1] scale. For benefit criteria, where higher values are desirable, such as Tests Per Capita or Vaccinations Per Capita:

(Equation 12) $$x_{ij}^{\prime }=\frac{x_{ij}-\text{mi}n_{j}\left(x_{ij}\right)}{\text{ma}x_{j}\left(x_{ij}\right)-\text{mi}n_{j}\left(x_{ij}\right)}$$

For cost criteria, where lower values are desirable, such as Mortality Rate or Cases Per Capita:

(Equation 13) $$x_{ij}^{\prime }=\frac{\text{ma}x_{j}\left(x_{ij}\right)-x_{ij}}{\text{ma}x_{j}\left(x_{ij}\right)-\text{mi}n_{j}\left(x_{ij}\right)}$$

This normalized matrix serves as the Criteria-to-Alternatives submatrix in the Analytic Network Process.

2 Criteria-to-Criteria Matrix Construction: Entropy weights $w_{j}$, computed from the normalized matrix quantify variability, with higher weights indicating greater influence, such as for Tests Per Capita. The C-C matrix models interdependencies by setting entry $c_{jk}=w_{k}∕w_{j}$ if the correlation between criteria j and k exceeds 0.20, otherwise 0. The 0.20 correlation threshold for the C-C matrix aligns with guidelines for moderate thresholds in correlation network analysis to capture meaningful interdependencies while minimizing noise.^42^ Each column is then normalized to sum to 1, ensuring column-stochastic properties. This formulation assumes that criteria with higher weights exert proportional influence on correlated ones, such as testing’s variability affecting mortality, grounded in empirical correlations from feature selection.
3 Alternatives-to-Alternatives Matrix Construction: Average Sustainable Development Goals scores from 2020 to 2023 proxy country interdependencies, incorporating resilience metrics like healthcare and economic stability. The matrix sets entry $a_{pq}=\frac{s_{p}}{s_{q}}$, where s denotes the score for countries p and q, modeling influence from higher-resilience nations to others through aid or policy diffusion. Each column is normalized to sum to 1, maintaining column-stochastic consistency and replacing subjective assessments with standardized data.
4 Supermatrix Assembly: The unweighted supermatrix W combines the goal cluster, Criteria-to-Criteria submatrix, Alternatives-to-Alternatives submatrix, Criteria-to-Alternatives submatrix, and Alternatives-to-Criteria submatrix, using the transpose to account for bidirectional feedback, such as country outcomes affecting reproduction rates.
5 Supermatrix Computation and Ranking: Cluster weights, derived from entropy averages, weight the supermatrix, ensuring column-stochastic properties. The matrix is raised to powers until convergence, with a tolerance of 1e-9 and a maximum of 10,000 iterations. Limit priorities from the alternatives column generate country rankings.
  The entropy method produces weights reflecting variability, while the Analytic Network Process incorporates these into network rankings, distinguishing their roles.

### Quantification and statistical analysis

All quantitative analyses were performed in Python (version 3.9+) using scikit-learn, pandas, and NumPy. Convergence of the ANP supermatrix was verified with a tolerance of 1 × 10^−9^ and a maximum of 10,000 iterations; convergence was achieved within 12 iterations. Correlation thresholds for Criteria-to-Criteria relationships were set at 0.20 to capture moderate interdependencies. Feature importance validation was conducted with a Random Forest regression model (80/20 train-test split), achieving *R*^2^ = 0.87. Random Forest importance values below 0.01 were discarded as noise. Hierarchical clustering of country rankings employed Ward’s method with Euclidean distance. All preprocessing and feature transformations (per capita adjustments, mortality rate computation, imputation) were standardized to ensure reproducibility.

## Acknowledgments

The authors wish to dedicate this work to the memory of Dr. Majid Khademati, whose visionary contributions to the field and invaluable guidance greatly influenced this study. His mentorship, insights, and encouragement continue to inspire our research. We honor his legacy and extend our deepest respect and gratitude.

## Author contributions

A.S. contributed to conceptualization, methodology, data curation, formal analysis, and writing – original draft. A.B. contributed to conceptualization, writing – review & editing, supervision, and validation. Both authors read and approved the final manuscript.

## Declaration of interests

The authors declare no competing financial or nonfinancial interests. The authors received no specific external funding for this study. The authors confirm transparent reporting of the use of AI-assisted language tools (ChatGPT) for editing support as noted above.

## Supplemental information

Table S1. Comprehensive ranking of countries using the ANP framework
